# Analysis of dynamically stable patterns in a maze-like corridor using the Wasserstein metric

**DOI:** 10.1038/s41598-018-24777-2

**Published:** 2018-04-23

**Authors:** Ryosuke Ishiwata, Ryota Kinukawa, Yuki Sugiyama

**Affiliations:** 1 Department of Complex Systems Science, Graduate School of Information Science, Nagoya University, Furo-chou, Chikusa-ku, Nagoya, Aichi 464-8601 Japan; 2 Present Address: Silicon Linux Corporation 1-7-5, Osu, Naka-Ku, Nagoya, Aichi 460-0011 Japan

## Abstract

The two-dimensional optimal velocity (2d-OV) model represents a dissipative system with asymmetric interactions, thus being suitable to reproduce behaviours such as pedestrian dynamics and the collective motion of living organisms. In this study, we found that particles in the 2d-OV model form optimal patterns in a maze-like corridor. Then, we estimated the stability of such patterns using the Wasserstein metric. Furthermore, we mapped these patterns into the Wasserstein metric space and represented them as points in a plane. As a result, we discovered that the stability of the dynamical patterns is strongly affected by the model sensitivity, which controls the motion of each particle. In addition, we verified the existence of two stable macroscopic patterns which were cohesive, stable, and appeared regularly over the time evolution of the model.

## Introduction

There is myriad of collective motions arising from groups of living organisms such as cells, animals, and human beings. Their underlying dynamics show adaptive responses to different aspects including physical, survival, and social interactions. For instance, patterns can emerge from cell migrations, the group formation in flock of birds or school of fish, and pedestrian flow in restricted passages within buildings^1–11^. These spontaneously emerging phenomena have been investigated from a physical perspective. Specifically, this type of motion can be modelled as a dissipative system with asymmetric interactions. In addition, this type of system does not satisfy both the action–reaction principle and the momentum conservation^7,12–15^. In addition, each moving unit in these systems acts as a self-driven particle, which can represent an independent living organism. Thus, these characteristics endow collective motion generated from dissipative systems with the ability to describe the group motion of living organisms. To investigate this collective motion, we use the two-dimensional optimal velocity (2d-OV) model^16–18^. In fact, the optimal velocity (OV) can represent the motion patterns of dissipative systems, where each particle aims to preserve an adequate velocity depending on its distance to neighbouring particles. For instance, the one-dimensional OV model, where particles reproduce a moving cluster, can be used to identify a traffic congestion^19^ and several related properties^20^. Likewise, the 2d-OV model can describe a variety of macroscopic patterns formed by moving particles, which can emulate the collective motion of living organisms^14,16^. For this model, Nakayama and Sugiyama^14,16^ have identified many types of dynamical patterns that depend on several parameters. The resulting macroscopic patterns exhibited by collective motion are promoted by asymmetric interactions.

**Figure 1:**
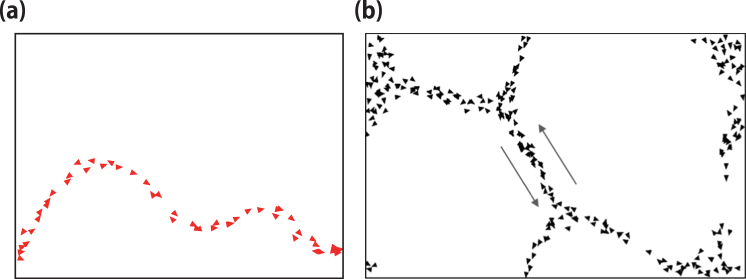
Patterns formed by particles using the 2d-OV model. Each triangle represents a particle, with its direction indicated by the tip. The black thin lines indicate periodic boundaries. (**a**) String-like pattern where particles move following a slow twisting. (**b**) Pattern resembling a minimum Steiner tree. The arrows indicate the formation of a counter flow.

The interaction between particles in the 2d-OV model can be either attractive or repulsive, depending on the distance among them. When particles show only attractive interaction, they tend to create string-like patterns. These patterns preserve their shape for a long time, and the particles move following a slow twisting (see panel (a) in Fig. 1)^16,21^. When a string-like pattern breaks into two patterns, they immediately adopt the same string-like characteristic, and when two string-like patterns break each other due to a collision, the particles in the OV model immediately reconstruct another string-like pattern.

These properties imply that a string-like pattern induces a continuous degree of freedom for deformation. For instance, the particles can form a network structure similar to a minimum Steiner tree (see panel (b) in Fig. 1)^14^. The network structure formed by the particles in the 2d-OV model is also similar to that formed by true slime moulds^22^. In addition, Nakagaki *et al*.^23^ and Tero *et al*.^24^ performed experiments to find the shortest paths that connect with two food sources through a maze using true slime moulds. Through these experiments, they investigated the adaptability induced by swarm intelligence.

In this paper, we attempt to perform similar experiments through the simulation of collective motion using the 2d-OV model. Specifically, we show that particles in the 2d-OV model exhibit optimal collective motions with neither a leader nor instructions for adapting to their physical environments. Based on simulation results, we explore the properties and mechanism that allow to understand adaptability in collective motion. These outcomes might provide an operative basis to understand phenomena such as collective motions and the induction of pedestrian evacuations.

First, we perform a numerical simulation of the 2d-OV model in a simple maze-like corridor. As a result, we show that macroscopic string-like patterns emerge for particles, which move as a queue that transverses the shortest paths.

Then, to investigate the time evolution of macroscopic patterns, we map the patterns using the Wasserstein metric space. This mapping allows the construction of a low-dimensional ‘similarity space’ to distinguish among different patterns. In addition, we illustrate the time evolution of the patterns in this space. Finally, we evaluate both the number of occurrences of the patterns and the sensitivity dependence of pattern stability.

## Methods

### The 2d-OV model with particle interaction

In this section, we provide a brief review on the 2d-OV model and the interaction between neighbouring particles. The 2d-OV model describes self-driven particles moving to maintain an OV that depends on their distance to neighbouring particles. We assume that *N* point particles move in the two-dimensional space. The equation of motion for the *j*-th particle (*j* = 1, 2, …, *N*) is expressed as

1 $${\ddot{\overrightarrow{x}}}_{j}(t)=a[{\overrightarrow{V}}_{s,j}+\sum _{k}\overrightarrow{V}({\overrightarrow{r}}_{kj}(t),{\dot{\overrightarrow{x}}}_{j}(t))-{\dot{\overrightarrow{x}}}_{j}(t)],$$

2 $$\overrightarrow{V}({\overrightarrow{r}}_{kj},{\dot{\overrightarrow{x}}}_{j})=(1+\,\cos \,{\theta }_{kj})f({r}_{kj}){\overrightarrow{n}}_{kj},$$

3 $$f({r}_{kj})=\alpha [\tanh \,\beta ({r}_{kj}-b)+c],$$

where ${\overrightarrow{x}}_{j}=({x}_{j},{y}_{j})$ is the position of the *j*-th particle; ${\overrightarrow{r}}_{kj}\equiv {\overrightarrow{x}}_{k}-{\overrightarrow{x}}_{j}$ and ${r}_{kj}\equiv |{\overrightarrow{r}}_{kj}|$ are the relative position and distance, respectively, between the *k*-th and *j*-th particles; ${\overrightarrow{n}}_{kj}\equiv {\overrightarrow{r}}_{kj}/{r}_{kj}$ is the normalised vector of ${\overrightarrow{r}}_{kj}$; *θ*_*kj*_ is the angle between the velocity of the *j*-th particle, ${\overrightarrow{x}}_{j}$, and relative position ${\overrightarrow{r}}_{kj}$; *a* represents the sensitivity, which determines the response strength; $\overrightarrow{V}({\overrightarrow{r}}_{kj},{\dot{\overrightarrow{x}}}_{j})$ is the asymmetric interaction of the *j*-th with the *k*-th particle; (1 + cos*θ*_*kj*_) indicates that a particle is more sensitive to particles in front than to those behind of it; *f*(*r*_*kj*_) indicates the type of interaction, with *f* < 0 and $f > 0$ representing repulsive and attractive interactions, respectively; *c* determines the distance of influence for repulsive and attractive interactions by −1 ≤ *c* ≤ 1^18^, which we set to 1 given that we only consider attractive interactions; *α* determines the interaction strength; *β* is the gradient at inflection point *b*; and *V&vec;*_*s*,*j*_ is a velocity term that guarantees particle self-motion in the absence of interactions^17,18,25^. Note that sensitivity *a* is expressed as the inverse of time and represents the relaxation rate. We set *α* = 0.25, *β* = 4, and ${\overrightarrow{V}}_{s,j}=0.75\,{\dot{\overrightarrow{x}}}_{j}/|{\dot{\overrightarrow{x}}}_{j}|$. The selection for velocity ${\overrightarrow{V}}_{s,j}$ first appeared in^16^, and causes the OV to be ${\overrightarrow{V}}_{s,j}+{\sum }_{k}\overrightarrow{V}({\overrightarrow{r}}_{kj},{\dot{\overrightarrow{x}}}_{j})$.

**Figure 2:**
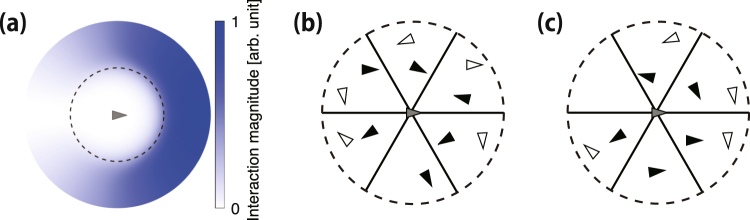
Interaction magnitude around a particle and selection of the nearest neighbours for interaction. Each triangle represents a particle, with its direction indicated by the tip. In panel (a), the highest interaction magnitude is indicated by the darkest colour. We calculated the interaction magnitude from $\overrightarrow{V}({\overrightarrow{r}}_{kj},{\dot{\overrightarrow{x}}}_{j})$, and inflection point *b* is indicated by the dashed circle. In panels (b) and (c), the dashed circle indicates the interaction range limit. The range is divided into 6 equal regions delimited by the solid lines. In each region, the black triangle indicates the nearest neighbour to the focal particle in the centre.

In panel (a) of Fig. 2, we show the interaction magnitude at the surroundings of a particle. Panels (b) and (c) illustrate the process to determine the interacting particles with respect to the focal particle located in the centre of the graph. First, we consider all the neighbouring particles within the interaction range of the focal particle. This range is fixed for every particle in the model and subsequently divided into 6 regions. Finally, we select the nearest neighbour (black triangle). Thus, each particle interacts with at most the 6 nearest neighbours as shown in panel (b) of Fig. 2, whereas panel (c) shows a case where only the 5 nearest neighbours are considered.

### Maze-like corridor structure and simulation parameters for particle motion

**Figure 3:**
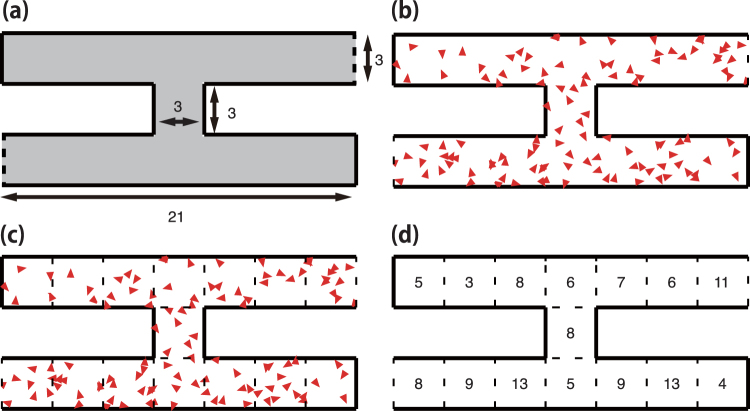
Maze-like corridor and particle distributions. Each triangle represents a particle, with its direction indicated by the tip. The solid lines represent elastic walls, and the two dashed lines are connected gates that form a periodic boundary, where particles pass through. When a particle collides with the wall, it reflects elastically. Particles can move within the grey region indicated in panel (a). (**b**) Initial configuration for the particles. (**c**) Example of particle distribution. Each triangle represents a particle, with its direction indicated by the tip. (**d**) Distribution represented by the number of particles contained in each cell.

We consider the simple maze-like corridor shown in panel (a) of Fig. 3 to evaluate the 2-d OV model. The corridor consists of two parallel sections and a connecting passage. In addition, the corridor is enclosed by elastic walls, represented by the solid lines, and it has two gates, represented by the dashed lines. The two gates are connected establishing a periodic boundary. Hence, the corridor has two dead ends at the upper-left and lower-right positions. The width and length of the corridor are 3 and 21, respectively, whereas the connecting passage is a square with sides having a length of 3.

We used the following parameters for the 2d-OV model: the number of particles was *N* = 115, the distance of the interaction range was set to 2, the inflection point of function tanh, *b*, to 1, and sensitivity *a* to 20. For the numerical simulations, we used an Euler scheme with the timestep of 0.001 and obtained the coarse data with a relative large timestep of 0.75. In addition, we selected a sensitivity value such that the right-hand side of equation (1) was smaller than 1. To start the simulation, we randomly distributed the particles in the maze-like corridor, as seen in panel (b) of Fig. 3, and randomly set each component of initial velocity from the interval (−0.1, 0.1).

### Wasserstein metric to analyse macroscopic patterns in collective motion

In this section, we introduce the mathematical approach to analyse the time evolution of macroscopic patterns in our simulation scenario. First, we express a macroscopic pattern by the distribution of particles in the maze-like corridor. Next, we introduce the Wasserstein metric to distinguish between a pair of macroscopic patterns. We call this approach the similarity measure between two macroscopic patterns. Finally, by using this similarity measure, we construct the corresponding metric space that reduces the dimensionality of the original metric space to clearly visualise the deformation process.

We define the distribution of particles corresponding to a macroscopic appearance at a given time as follows. We divide the maze-like corridor into cells (Fig. 3, panel (c)) and count the number of particles within each cell (panel (d)). Hence, the distribution of a cell is defined by the number of particles contained in it. By repeating this process over time, we can obtain the evolution of the particle distribution throughout the deformation process.

Next, we introduce the similarity measure between macroscopic patterns based on those distributions by using the Wasserstein metric^26–29^ (details on the Wasserstein metric are provided in Supplementary Information A). Let us suppose that we transform a pattern *μ* into another *ν* by transporting the particles. Patterns *μ* and *ν* have a given the numbers of particles in each cell. The number of particles in the *i*-th (or *j*-th) cell of pattern *μ* (or *ν*) is denoted by *μ*_*i*_ (or *ν*_*j*_). A transportation plan *f* is determined by every *f*_*ij*_ representing the number of particles transported from *μ*_*i*_ to *ν*_*j*_. In addition, *c*_*ij*_ represents the transportation cost of a particle from the *i*-th to the *j*-th cell. If we suitably define the transportation cost, we can find the minimum transportation cost among several transportation plans. Then, we determine the similarity between patterns *μ* and *ν* as the minimum transportation cost, *C*(*μ*,*ν*), given by

4 $$C(\mu ,\nu )=\mathop{{\rm{\min }}}\limits_{{f}_{ij}}\sum _{i,j}\,{f}_{ij}\,{c}_{ij},$$

which must satisfy constraints ∑_*j*_ *f*_*ij*_ = *μ*_*i*_ and ∑_*i*_*f*_*ij*_ = *ν*_*j*_. We selected the Euclidean distance between the *i*-th and *j*-th cells to represent cost *c*_*ij*_. Then, we calculate *C*(*μ*, *ν*) among every pair of patterns for all times throughout the deformation process obtained from the simulation. We introduce affinity matrix *B* with entries given by similarity measures as *B*_*m*,*n*_ = *C*(*μ*(*t*_*m*_),*μ*(*t*_*n*_)), where *μ*(*t*_*m*_) and *μ*(*t*_*n*_) represent the patterns at times *t*_*m*_ and *t*_*n*_, respectively. Therefore, we can compare all pairs of patterns using the similarity measures in this matrix.

Finally, we construct the metric space using the similarity measures. In this metric space, each macroscopic pattern at a given time corresponds to a point. Moreover, we reduce the dimensionality of this metric space using the classical multidimensional scaling technique^30^. This technique allows to find the coordinate of a point on the Euclidean metric space corresponding to a point given in another metric space. To apply the technique, we calculate the eigenvalues and corresponding eigenvectors from affinity matrix *B*. Using the two largest eigenvalues and their corresponding eigenvectors, we construct a two-dimensional space and obtain the coordinates of points representing the macroscopic patterns. As a result, each pattern at a given time throughout the deformation process is represented by a point in the plane. Hence, we can map the deformation process of macroscopic patterns to a trajectory in the plane (we calculated the trajectory in a two-dimensional Euclidean space by using the MASS package from the R language^31^). In this paper, we call the plane representing patterns as the Wasserstein metric space.

## Results

### Deformation evolution of macroscopic patterns

**Figure 4:**
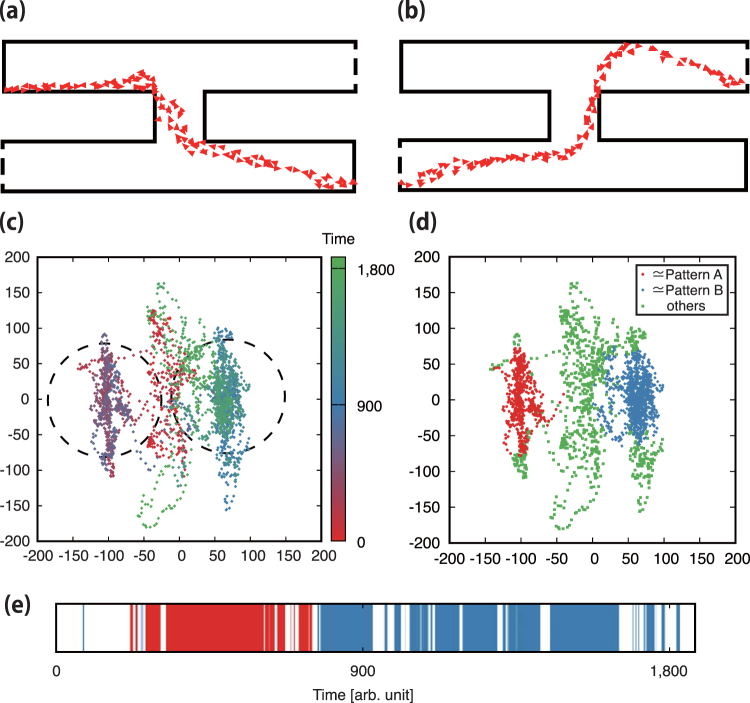
Particle distribution within cells and patterns mapped into the Wasserstein metric space. (**a**) Particles move back and forth between the upper-left and bottom-right dead ends. (**b**) Particles pass through the gates with motion in two directions. (**b**) Macroscopic pattern evolution over time for sensitivity *a* = 20 in the two-dimensional Wasserstein metric space. The dashed circles delimit clusters of similar patterns. Patterns in both the left and right clusters exhibit the structure in panels (a) and (b), respectively. (**d**) Pattern classification. Red and blue points represent patterns A and B, respectively, whereas green points do no exhibit any string-like pattern. (**e**) Pattern evolution over time throughout the deformation process. Red and blue bars represent particles exhibiting patterns A and B, respectively, and blank areas represent no string-like pattern.

At the beginning of the simulation, the particles formed several moving clusters. These clusters tended to combine as they approached. After the first relaxation time, the collective motion of particles converged to the string-like pattern that goes back and forth between the two dead ends, as shown in panel (a) of Fig. 4. At moments, the string-like pattern exhibited fluctuations. Then, after the second relaxation time, the particles formed another string-like pattern passing through the two gates as shown in panel (b). These two string-like patterns showed a bidirectional flow of particles, and each was maintained over a long time. This behaviour suggests that either of the two patterns represents the shortest path solution in the maze-like corridor.

Furthermore, we noted that the number of particles affects the formation of macroscopic patterns. When the number of particles is very small, they move randomly and form no macroscopic pattern. In contrast, when this number is very large, the particles almost fully occupy the corridor. Therefore, an appropriate number of particles should be selected to achieve the formation of patterns that reflect the optimal path in the maze-like corridor. This selection was suggested in a preliminary work^32^.

For any initial configuration with the appropriate number of particles, similar string-like patterns are formed and maintained. We found that the particles can form two quasi-stable macroscopic patterns traversing the shortest paths in the considered maze-like corridor. Given the quasi-stability of these patterns, we further investigated the deformation process and pattern characteristics by using the Wasserstein metric.

### Pattern evolution in the Wasserstein metric space

Panel (c) of Fig. 4 illustrates the time evolution of the patterns generated from the scenario depicted in panel (b) of Fig. 3 and panels (a) and (b) of Fig. 4. Panel (c) of Fig. 3 shows that most patterns, which correspond to points in the graph, are mapped into the left and right regions of the plane, and correspond to the stable string-like patterns in panels (a) and (b) of Fig. 4, respectively. The point accumulations in the two regions suggest the stability of the string-like patterns.

Based on the two stable string-like patterns, we characterise the deformation process of the pattern as follows. First, we choose as reference the two patterns, namely, pattern A, shown in panel (a) of Fig. 4, and pattern B, shown in panel (b). Next, we define a region that describes both patterns in the Wasserstein metric space, which are shown as the circles in panel (c) of Fig. 4, whose radius *R* was fixed to 80. Then, we classify the macroscopic patterns as belonging to pattern A, pattern B, or neither. Specifically, if pattern *μ* satisfies *C*(*μ*,*A*) ≤ *R* or *C*(*μ*,*B*) ≤*R*, then *μ* is classified as pattern A or pattern B, respectively. Panel (d) of Fig. 4 highlights the classified results in the Wasserstein metric space. In Supplementary Information B, we detail the classification of macroscopic patterns using model-based clustering, which retrieves similar results as the classification presented here.

In panel (e) of Fig. 4, we represent patterns A and B as vertical bars coloured in red and blue, respectively, distributed along a linear time scale, whereas the blank areas represent no string-like pattern. Hence, regions of this scale sharing the same colour indicate that the corresponding pattern is maintained over time. For instance, pattern A is formed and maintained at an early stage, whereas pattern B is subsequently formed and maintained for a longer time than pattern A. Overall, the two string-like patterns are consistently maintained over time, thus indicating certain amount of stability.

### Sensitivity dependence for macroscopic patterns

In the 2d-OV model, sensitivity value *a* affects the pattern formation of particles^14,16,18,32^. For our study, we evaluated the relation between sensitivity and both the emerging patterns and their stability. This evaluation was performed in the Wasserstein metric space.

#### Emerging macroscopic patterns

**Figure 5:**
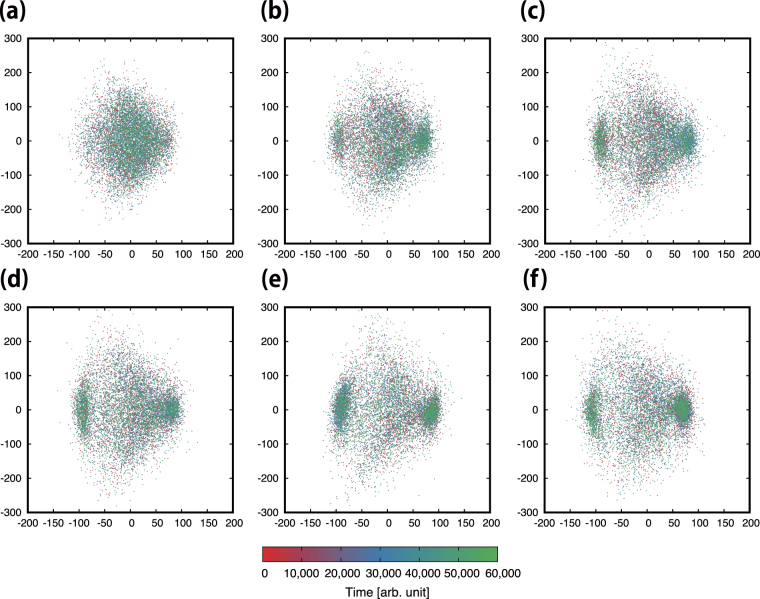
Macroscopic pattern evolution represented by points in the two-dimensional Wasserstein metric space. The colour scale indicates time evolution. Panels (a) through (**f**) represent the pattern evolution for *a* = 10, 15, 20, 25, 30 and 35, respectively. Given the longer simulation time, it may be more difficult to see the pattern evolution than in panel (c) of Fig. 4. The patterns in the left and right clusters are similar to the string-like patterns A and B, respectively.

We investigated the sensitivity dependence of the emerging patterns in the deformation process considering 6 different sensitivity values: *a* = 10, 15, 20, 25, 30, and 35. We performed the corresponding simulations over a long time and used the coarse sampling data with a long timestep of 6.0. As a result, we found that the same stable string-like patterns, namely, patterns A and B, appear regardless of the sensitivity value. These patterns are represented in Fig. 5 by the two accumulations of points in the separated regions of the Wasserstein metric space. In the cases *a* = 20, 25, and 30 (see panels (c), (d), and (e)), the accumulation of points in the left and right regions of the space correspond to the string-like patterns A and B. In contrast, the accumulation is not clear in the cases *a* = 10 and 15 (see panels (a) and (b)). We found no other macroscopic pattern except for the two string-like patterns A and B.

**Figure 6:**
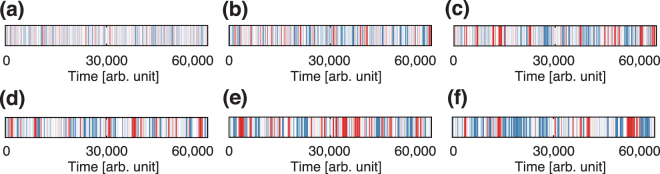
Pattern evolution throughout the deformation process. Panels (a) though (**f**) represent the processes for *a* = 10, 15, 20, 25, 30 and 35, respectively. The red and blue vertical bars indicate the instants when particles exhibit patterns A and B, respectively.

Next, in Fig. 6 we illustrate the pattern variation throughout the deformation process depending on the type of string-like pattern, either pattern A or B. We found that with sensitivity values *a* = 20, 25, 30, and 35 (panels (c), (d), (e), and (f), respectively), the string-like patterns are formed more often and preserved for a longer time than with the other sensitivity values, *a* = 10 and 15 (panels (a) and (b), respectively).

#### Stability of string-like patterns

From the behaviour illustrated in Fig. 6, we investigated the sensitivity dependence on the stability of the string-like patterns. We estimated the stability based on the number of occurrences and timespan of the string-like patterns. Let us denote the number of occurrences for patterns A and B as *F*_*A*_(*l*) and *F*_*B*_(*l*), respectively, where *l* is the timespan. To quantify the number of occurrences for each timespan, we determined the number of successive bars of the same colour, which corresponds to a given pattern, to represent the duration of a pattern by the number of timesteps.

Using number of occurrences and timespan, we calculated the average timespans, *W*_*A*_ and *W*_*B*_, as

$${W}_{A}:=\frac{\sum _{l\ge 1}l\,{F}_{A}(l)}{\sum _{l\ge 1}{F}_{A}(l)},$$

$${W}_{B}:=\frac{\sum _{l\ge 1}l\,{F}_{B}(l)}{\sum _{l\ge 1}{F}_{B}(l)}\mathrm{.}$$

**Figure 7:**
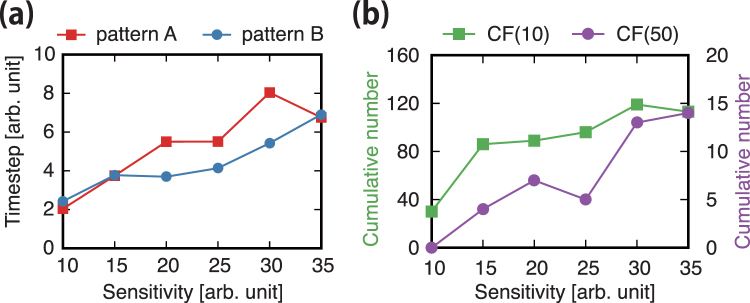
Average timespans and cumulative numbers of occurrences for the string-like patterns. (**a**) Average timespans of string-like patterns according to sensitivity *a*. (**b**) Cumulative number of occurrences with short (i.e. *CF*(10)) and long (i.e. *CF*(50)) timespans according to sensitivity *a*.

We show the average timespan in panel (a) of Fig. 7. The variation of the average timespan according to the sensitivity is similar for patterns A and B. The average timespans show their highest values at sensitivities of 30 and 35, which thus retrieve the highest stability of the string-like patterns.

In addition, we calculated another quantity to investigate the stability of the string-like patterns by the number of occurrences. We define the cumulative number of occurrences for the two string-like patterns as

5 $$CF(\tilde{l}):=\sum _{l\ge \tilde{l}}{F}_{A}(l)+\sum _{l\ge \tilde{l}}{F}_{B}(l),$$

where $\tilde{l}$ is a timespan threshold (the variation of $CF(\tilde{l})$ according to $\tilde{l}$ is shown in Fig. S2 from Supplementary Information). Hereafter, we ignore the difference between patterns A and B for convenience (statistical results are shown in Fig. S3 from Supplementary Information).

Using the cumulative number of occurrences, we can estimate the cohesion of string-like patterns. To investigate the sensitivity dependence of this property, we show the cumulative number of occurrences for a short (i.e. *CF*(10)) and a long (i.e. *CF*(50)) timespan, for sensitivity values *a* = 10, 15, 20, 25, 30, and 35 in panel (b) of Fig. 7. The string-like patterns are more cohesive for a sensitivity in the middle range of values. Specifically, the string-like patterns are more cohesive for sensitivity values of 30 and 35.

Consequently, both the average timespan, shown in panel (a) of Fig. 7, and the pattern cohesion, shown in panel (b), have their highest values in the same sensitivity range around 30 (statistical results are detailed in Supplementary Information D).

In summary, this section details the sensitivity dependence of the emerging patterns along the deformation process, considering different sensitivity values. We found that the stable string-like patterns, namely, patterns A and B, are represented by two separated regions in the Wasserstein metric space (Fig. 5). In addition, we found that there no other macroscopic pattern emerges except for these two string-like patterns. In addition, the sensitivity dependence on the pattern stability is addressed. As seen in Figs 6 and 7, we found that the stability and cohesiveness of the string-like patterns have their highest values for sensitivity *a* around 30. In Supplementary Information E, we show that the same relations between sensitivity and cohesiveness are obtained using recurrence quantification analysis^33^.

## Discussion

The main outcomes from this study are summarised as follows. We investigated the 2d-OV model, which represents a simple dissipative system with asymmetric interactions. We used this model to study the adaptability of collective motion usually seen in groups of living organisms. In addition, we performed numerical simulations in a maze-like corridor. We found the generation of two string-like patterns (see panels (a) and (b) of Fig. 4) that transverse the shortest paths in the corridor and are maintained for a long time. Next, to both investigate the deformation process and evaluate the stability of the generated macroscopic patterns, we constructed the reduced-dimension Wasserstein metric space, which allows to represent the difference between macroscopic patterns based on the similarity measure. In this space, a macroscopic pattern in the corridor is represented by a point, and the deformation process is given by a trajectory. Using this mathematical technique, we verified that two stable patterns exist (see panel (c) of Fig. 4) and evaluated their stability in relation to the sensitivity of the 2d-OV model. We also confirmed that it is necessary an appropriate number of particles in the model to establish the patterns traversing the shortest paths in the maze-like corridor. Moreover, we observed the long timespan of these patterns for large sensitivity values from the analysis based on Fig. 6. For the large sensitivity values, the particle motion is strongly affected by the attractive interaction. The attractive interaction arises optimal collective motions with neither a leader nor instructions for adapting to their physical environments. Such a effect of attractive interaction to the adaptation is observed in the previous studies^1,34^. In addition, we confirmed the formation of a shortest-path pattern in a more complicated maze-like corridor, as shown in Fig. S9 of Supplementary Information. This pattern is similar to the construction obtained using true slime moulds^23^.

In our investigation, we found that the time evolution of the considered non-equilibrium and many-body system converges to localised regions in the Wasserstein metric space. From a statistical physics point of view, we consider that the deformation process might be expressed by a gradient flow of some potential defined in this space. Thus, we have the possibility to find such a potential using a few appropriate coarse-grained variables which span over this reduced-dimension space. Moreover, we suppose that the potential can be represented by some kind of thermodynamic potential for the steady-state solution of the system. We further suppose that this potential controls the stability of the macroscopic patterns like in an ‘energy landscape’^35–37^, as in the case of physical biology^38,39^.

In collective motions, the stability of macroscopic patterns may be organised according to the state degeneration. In fact, by considering a purely attractive interaction, the collective motion of the particles in the 2d-OV model formed a twisting string-like pattern, which implies that the pattern has a continuous degree of freedom for deformation. This property further implies the high degeneracy of the ground state in a thermodynamic potential. Given the degree of freedom in the ground state, the collective motion spontaneously forms an optimal flow pattern fitting to the boundary conditions of the maze-like corridor. We expect that this property in the dissipative system with asymmetric interactions represents a physical mechanism for the adaptability of collective motions.

## Electronic supplementary material

Supplementary information
