# Supplementary material for: Analysis of dynamically stable patterns in a maze-like corridor using the Wasserstein metric
Source: Sci Rep. 2018 Apr 23;8:6367. doi: 10.1038/s41598-018-24777-2 (PMC5913310; doi:10.1038/s41598-018-24777-2)
Supplement: Supplementary file 1 — Supplementary information [file 41598_2018_24777_MOESM1_ESM.pdf]

Analysis of dynamically stable patterns in a maze-like corridor using the Wasserstein metric: Supplementary information

Ryosuke Ishiwata[1,*], **Ryota Kinukawa**[1,2], **and Yuki Sugiyama**[1]

[1]Department of Complex Systems Science, Graduate School of Information Science, Nagoya University, Furo-chou, Chikusa-ku, Nagoya, Aichi, 4648601 Japan.
[2]Current address: Silicon Linux Corporation 1-7-5, Osu, Naka-Ku Nagoya, Aichi, 460-0011 Japan.
[*]ishiwata@phys.cs.i.nagoya-u.ac.jp

ABSTRACT

In this document, we detail the Wasserstein metric and model-based clustering for the maze-like patterns. Likewise, we show results on the number of occurrences and cumulative number of occurrences for string-like patterns, and on recurrence quantification analysis. Finally, we illustrate pattern formation in a more complicated maze-like corridor than that used for the analysis in our study.

A Wasserstein metric

The Wasserstein distance is defined as a solution for the optimal transportation problem[1,2], and it is also known as the Earth-mover distance in the field of information science[3]. The optimal transportation problem can be formulated in terms of the Wasserstein distance through a pair of measurable spaces, (X,μ) and (Y,ν), and a cost function, $c(x,y)$, where $x \in X$, $y \in Y$, and μ and ν are probability measures. In this study, we considered both X and Y to be the Euclidean plane. Hence, the plane and its elements are denoted by X and $\vec{x}_1, \vec{x}_2 \in X$, respectively. In addition, we set the cost function, $c(\vec{x}_1,\vec{x}_2)$, as the Euclidean distance defined by $c(\vec{x}_1,\vec{x}_2) := \|\vec{x}_1 - \vec{x}_2\|$. The Wasserstein distance between μ and ν is defined as

$$C(\mu,\nu) := \min_{\pi} \int_{X\times X} \mathrm{d}\pi(\vec{x}_1,\vec{x}_2)\, \|\vec{x}_1 - \vec{x}_2\|, \tag{1}$$

where π is a probability measure on product space $X \times X$ and satisfies

$$\begin{aligned} \mu(\vec{x}_1) &= \int_X \mathrm{d}x_2\, \pi(\vec{x}_1,\vec{x}_2), \\ \nu(\vec{x}_2) &= \int_X \mathrm{d}x_1\, \pi(\vec{x}_1,\vec{x}_2). \end{aligned}$$

Probability measure π is generally called a transportation plan. When a certain transportation plan π minimises $\int_{X\times X} \mathrm{d}\pi(\vec{x}_1,\vec{x}_2)\, \|\vec{x}_1 - \vec{x}_2\|$, π is the optimal plan.

B Classification of macroscopic patterns using the model-based clustering

For a typical dataset with $a = 20$, we classify each macroscopic pattern as belonging to one of three clusters. Panel (a) of Fig. S1 shows the application of a model-based clustering that is ellipsoidal model with

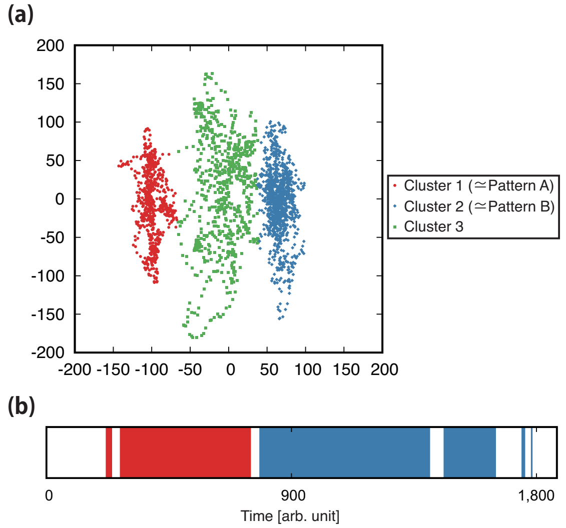

Figure S1. Model-based clustering. (a) Classification in the Wasserstein metric space over three clusters using an ellipsoidal model with varying volume, shape, and orientation. (b) Pattern classification distributed over a linear time scale. The red and blue bars indicate patterns classified into cluster 1 and cluster 2 over time, respectively, whereas the blank areas indicate patterns not corresponding to any cluster.

varying volume, shape, and orientation applying the 'mclust' function[4]. Then, we depict the classified patterns over a linear time scale, as shown in panel (b) of Fig. S1.

C Number of occurrences and cumulative number of occurrences for a string-like pattern

Fig. S2 shows the variation of cumulative number of occurrences $CF\left(\tilde{l}\right)$ for sensitivity $a = 10$, 15, 20, 25, 30, and 35. We repeatedly performed numerical simulations with different initial configurations, i.e. particles were randomly distributed and assigned random velocities in the maze-like corridor, to obtain 20 datasets per sensitivity value. Then, we calculated the similarity among every pair of patterns throughout the deformation process and classified them as pattern A, pattern B, or neither for every instant. Using these classification data, we calculated the ensemble average and standard deviation for the cumulative number of occurrences, $CF_A\left(\tilde{l}\right) := \sum_{l\geq\tilde{l}} F_A\left(l\right)$ and $CF_B\left(\tilde{l}\right) := \sum_{l\geq\tilde{l}} F_B\left(l\right)$. We show the results from this process in Fig. S3.

D Statistics on average timespan and cumulative number of occurrences

We performed long-time simulations and obtained datasets with the sensitivity values $a = 10$, 15, 20, 25, 30, and 35 and a short timestep $dt = 0.001$. Likewise, we used coarse sampling data with a large timestep of 6.0 and obtained 20 datasets per sensitivity value. Then, we calculated average timespan $W := W_A + W_B$, where $W_A := \frac{\sum_{l\geq 1} l F_A(l)}{\sum_{l\geq 1} F_A(l)}$, $W_B := \frac{\sum_{l\geq 1} l F_B(l)}{\sum_{l\geq 1} F_B(l)}$, and cumulative number of occurrences $CF\left(\tilde{l}\right) := \sum_{l\geq\tilde{l}} F_A\left(l\right) + \sum_{l\geq\tilde{l}} F_B\left(l\right)$ for each dataset. Finally, using 20 samples per sensitivity value, we applied the one-way analysis of variance with Tukey's post-hoc test, whose results are shown in Fig. S4.

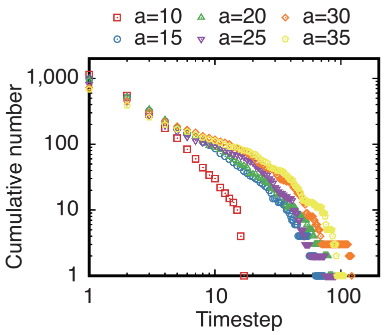

Figure S2. Cumulative number of occurrences $CF\left(\tilde{l}\right)$ for different timespans and sensitivity values.

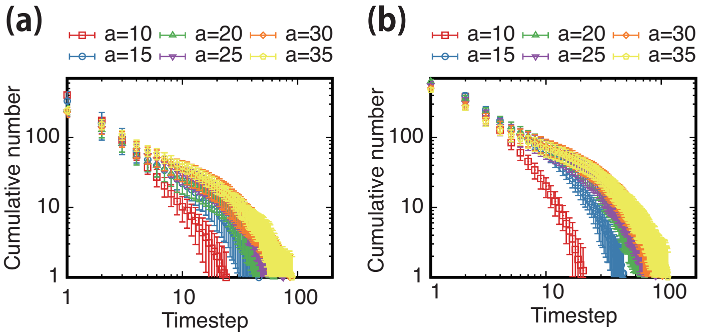

Figure S3. Ensemble averages for cumulative number of occurrences, (a) $CF_A\left(\tilde{l}\right)$ and (b) $CF_B\left(\tilde{l}\right)$ for different timespans and sensitivity values.

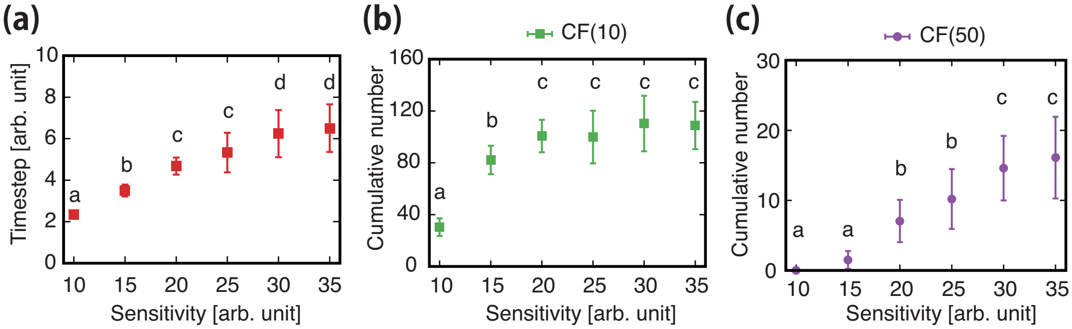

Figure S4. Statistical results of average timespan and cumulative number of occurrences for the macroscopic patterns. (a) Average timespan of patterns according to sensitivity a. Cumulative number of occurrences with (b) short (i.e. $CF(10)$) and (c) long (i.e. $CF(50)$) timespans. The data points represent mean $\pm$ SD. The letters indicate statistically significant differences (one-way analysis of variance with Tukey's post-hoc test; $p < 0.01$; $n = 20$).

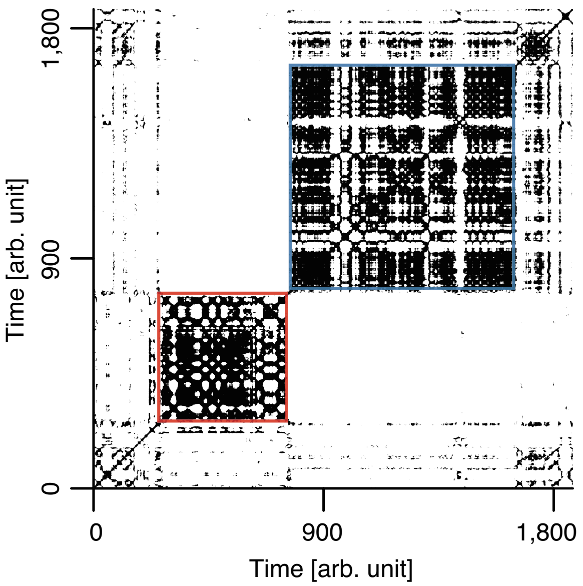

Figure S5. Recurrence plots of the Wasserstein metric with threshold $\varepsilon = 80$. The bottom left area (red rectangle) corresponds to pattern A, whereas the upper right area (blue rectangle) corresponds to pattern B.

E Recurrence plot and recurrence quantification analysis

Recurrence plot generates two-dimensional diagrams for visualising the recurrence of trajectories[5–7]. Suppose that a trajectory is given by time-sequential data x_m, where m represents a timestep ($m = 1, 2, \ldots, N$). The recurrence plot verifies the pairwise closeness among states. This closeness is defined as $R_{m,n} := \Theta(\varepsilon - B_{m,n})$, where Θ is the Heaviside function, ε is a threshold, and $B_{m,n}$ is the distance between states x_m and x_n. The resulting matrix, $R_{m,n}$, is the recurrence plot.

When trajectory x_m contains typical dynamical behaviours, a characteristic structure appears in the recurrence plot. Specifically, if a state does not vary over some time, which is known as laminar state, it appears as horizontal and vertical lines. To quantify this characteristic structure, Marwan et al.[6] proposed the recurrence quantification analysis for the vertical lines. Fig. S5 illustrates a recurrence plot from affinity matrix $B_{m,n} = C(\mu(t_m), \mu(t_n))$ of the dataset shown in Fig. 4 in the main text and threshold $\varepsilon = 80$. The filled areas represent sets of laminar states, which mostly correspond to patterns A (red rectangle) or B (blue rectangle).

Then, two measures are considered for recurrence quantification analysis, namely, laminarity LAM and trapping time TT. Laminarity is defined as the ratio between the recurrence points forming the vertical structures and all the recurrence points, and represents the occurrence of laminar states in the system:

$$LAM := \frac{\sum_{v=v_{\min}}^{N} v P(v)}{\sum_{v=1}^{N} v P(v)},$$

where $P(v)$ is the total number of vertical lines of the length v in the recurrence plot and $v_{\min}$ is a predefined minimal length. Trapping time is defined as the average length of vertical structures and represents the timespan of states:

$$TT := \frac{\sum_{v=v_{\min}}^{N} v P(v)}{\sum_{v=v_{\min}}^{N} P(v)}.$$

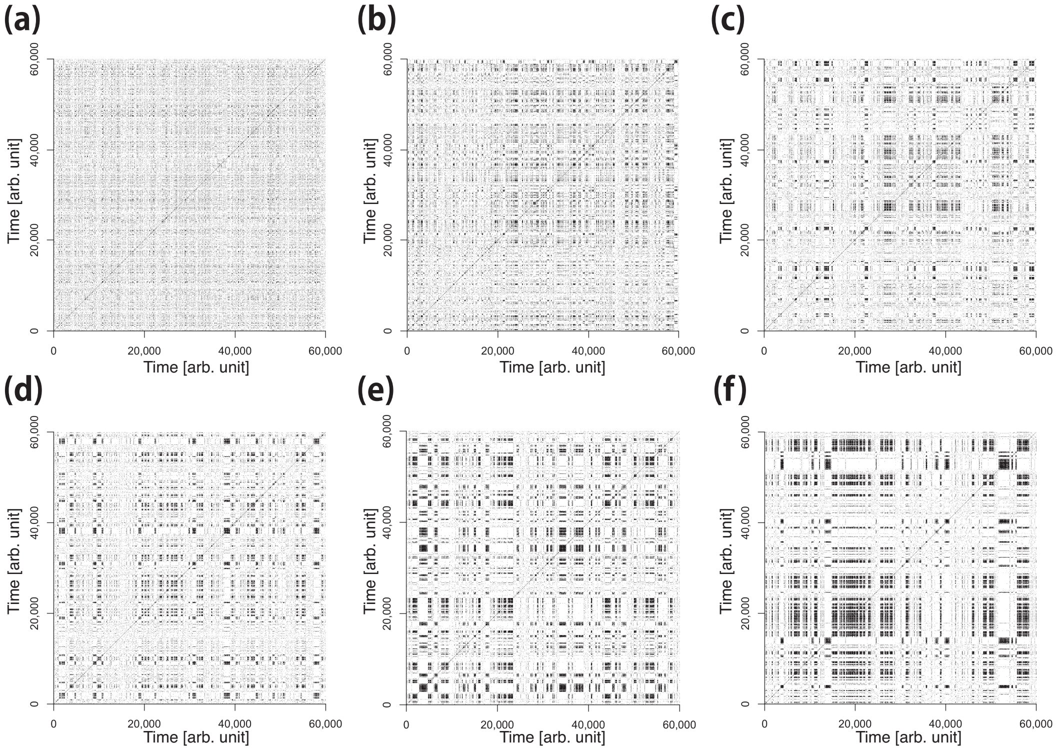

Figure S6. Recurrence plots of the Wasserstein metric with threshold $\varepsilon = 80$ for sensitivity $a = 10$, 15, 20, 25, 30, and 35 in panels (a) through (f), respectively.

We evaluated the sensitivity dependence of laminar states in the particle distributions by calculating the recurrence plots from the affinity matrix for the Wasserstein metric (equation (4) in the main text). Fig. S6 depicts matrix $R_{m,n} := \Theta(\varepsilon - B_{m,n})$, where $\varepsilon = 80$. Almost all the points in identifiable structures correspond to either pattern A or B (respectively, Fig. 4 panels (a) and (b) in the main text). Fig. S7 shows the LAM and TT outcomes for the dataset corresponding to Fig. 6, whereas Fig. S8 shows the statistical results considering 20 datasets at different sensitivity values. The figures show that both the trapping time and laminarity have their highest values for sensitivity a of 30 and 35.

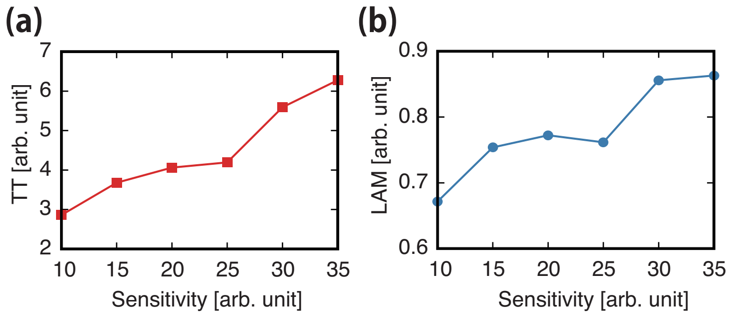

Figure S7. Recurrence quantification analysis. (a) Trapping time TT and (b) laminarity LAM according to sensitivity a.

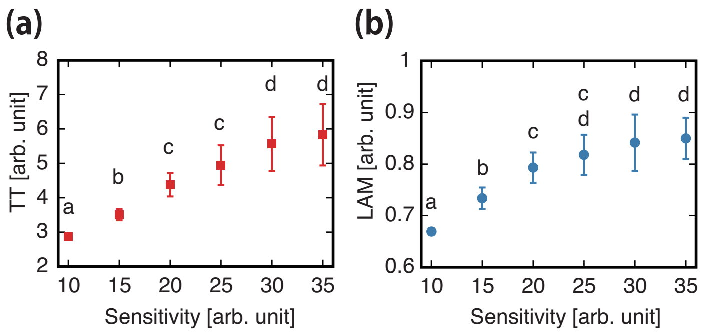

Figure S8. Statistical results of recurrence quantification analysis. (a) Trapping time TT and (b) laminarity LAM according to sensitivity a. The data points represent mean $\pm$ SD. The letters indicate statistically significant differences (one-way analysis of variance with Tukey's post-hoc test; $p < 0.01$; $n = 20$).

F String-like pattern in another maze-like corridor

Fig. S9 shows an emerging string-like pattern in a more complicated corridor than that used for our analysis, with $N = 250$ particles, and sensitivity $a = 25$.

References

1. Villani, C. *Topics in Optimal Transportation* (American Mathematical Soc., 2003).
2. Villani, C. *Optimal transport : old and new*. Die Grundlehren der mathematischen Wissenschaften (Springer, 2009).
3. Rubner, Y., Tomasi, C. & Guibas, L. J. *A metric for distributions with applications to image databases* (IEEE, 1998).
4. Scrucca, L., Fop, M., Murphy, T. B. & Raftery, A. E. mclust 5: Clustering, Classification and Density Estimation Using Gaussian Finite Mixture Models. *The R journal* **8**, 289–317 (2016).
5. Eckmann, J. P., Oliffson Kamphorst, S. & Ruelle, D. Recurrence plots of dynamical systems. *Europhys. Lett.* **4**, 973–977 (1987).
6. Marwan, N., Wessel, N., Meyerfeldt, U., Schirdewan, A. & Kurths, J. Recurrence-plot-based measures of complexity and their application to heart-rate-variability data. *Phys. review. E, Stat. nonlinear, soft matter physics* **66**, 026702 (2002).
7. Marwan, N., Carmen Romano, M., Thiel, M. & Kurths, J. Recurrence plots for the analysis of complex systems. *Phys. Reports* **438**, 237–329 (2007).

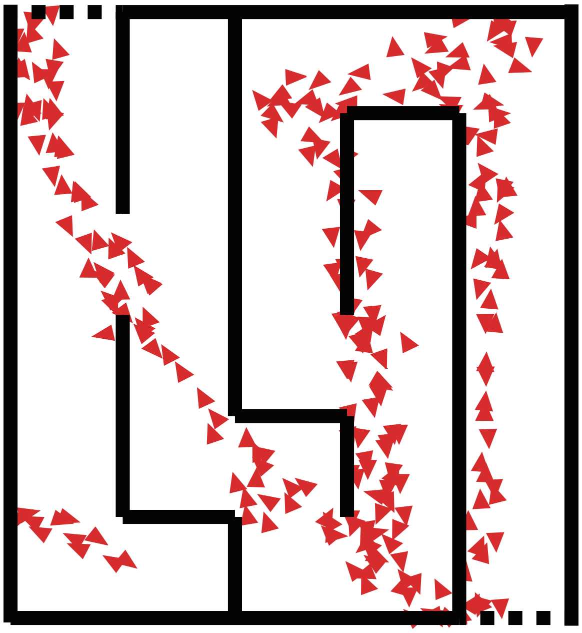

Figure S9. String-like pattern formed in a more complicated corridor than that used for our analysis. Each triangle represents a particle, with its direction indicated by the tip. The solid lines represent elastic walls, and the two dashed lines are connected gates that form a periodic boundary.
